# Changes in Ultrastructure and Cytoskeletal Aspects of Human Normal and Osteoarthritic Chondrocytes Exposed to Interleukin-1β and Cyclical Hydrostatic Pressure

**DOI:** 10.3390/ijms161125936

**Published:** 2015-10-30

**Authors:** Nicola Antonio Pascarelli, Giulia Collodel, Elena Moretti, Sara Cheleschi, Antonella Fioravanti

**Affiliations:** 1 Department of Medicine, Surgery and Neuroscience, Rheumatology Unit, University of Siena, Policlinico Le Scotte, Viale Bracci 1, Siena 53100, Italy; pascarelli@unisi.it (N.A.P.); saracheleschi@hotmail.com (S.C.); 2 Department of Molecular and Developmental Medicine, University of Siena, Siena 53100, Italy; giulia.collodel@unisi.it (G.C.); elena.moretti@unisi.it (E.M.)

**Keywords:** cytoskeleton, human chondrocytes, hydrostatic pressure, interleukin-1β, osteoarthritis, ultrastructure

## Abstract

The aim of this study was to examine the ultrastructure and cytoskeletal organization in human normal and Osteoarhritic (OA) chondrocytes, exposed to interleukin-1β (IL-1β) and cyclic hydrostatic pressure (HP). Morphological examination by transmission electron microscopy (TEM) and scanning electron microscopy (SEM) revealed differences between normal and OA chondrocytes at the nuclear and cytoplasmic level. IL-1β (5 ng/mL) induced a decrease of the number of mitochondria and Golgi bodies and a significant increase on the percentage of cells rich in vacuolization and in marginated chromatin. Cyclical HP (1–5 MPa, 0.25 Hz, for 3 h) did not change the morphology of normal chondrocytes, but had a beneficial effect on OA chondrocytes increasing the number of organelles. Normal and OA cells subjected to IL-1β and HP recovered cytoplasmic ultrastructure. Immunofluorescence (IF) examination of normal chondrocytes showed an actin signal polarized on the apical sides of the cytoplasm, tubulin and vimentin uniformly distributed throughout cytoplasm and vinculin revealed a punctuated pattern under the plasma membrane. In OA chondrocytes, these proteins partially lost their organization. Stimulation with IL-1β caused, in both type of cells, modification in the cytoskeletal organization; HP counteracted the negative effects of IL-1β. Our results showed structural differences at nuclear, cytoplasmic and cytoskeletal level between normal and OA chondrocytes. IL-1β induced ultrastructural and cytoskeletal modifications, counteracted by a cyclical low HP.

## 1. Introduction

Osteoarthritis (OA) is the most prevalent musculoskeletal joint disease directly involving the articular cartilage. Accumulating evidence suggest the pathogenetic role of some enzymes as matrix metalloproteinases (MMPs) and metalloproteinase with thrombospondin motif (ADAMTS) on joint destruction [1,2].

Interleukin-1β (IL-1β) plays a pivotal role on cartilage degradation processes. As a result of IL-1β effects, chondrocytes produce pro-inflammatory cytokines, chemokines, MMPs, and nitric oxide (NO). This cytokine can stimulate its own production, and inhibit the synthesis of extracellular matrix (ECM) [3,4,5]. Therefore, IL-1β is commonly used in *in vitro* studies to represent the circumstances leading to *in vivo* cartilage degradation [6]. The function of chondrocytes is also influenced by mechanical factors; under physiological conditions, articular cartilage is subjected to cycles of loading, which control the matrix through the metabolic activity of chondrocytes [7,8]. These loads alter the extracellular physical environment of the chondrocyte in a complex manner. Several *in vitro* studies demonstrated the important role of mechanical compression or hydrostatic pressure (HP) as a modulator of cartilage metabolism [9,10,11]. HP can also modify cellular morphology as demonstrated by transmission electron microscopy (TEM) and scanning electron microscopy (SEM) [11,12].

Further pathogenetic aspects of OA include modifications in the phenotype and cytoskeletal organization of chondrocytes [13]. The cellular cytoskeleton plays a critical role in the regulation of chondrocyte phenotype and in the physical interactions between chondrocytes and their ECM; it may, therefore, be involved in the process of mechanical signal transduction in articular cartilage [14]. Furthermore, cytoskeleton disruption in chondrocytes might be involved in OA pathogenesis [15]. The cytoskeleton of chondrocytes is made up of microfilaments formed by subunits of actin, tubulin microtubules, and intermediate filaments consisting of different protein subunits [16]. Actin filaments carry out a fundamental function in the control of cell shape, movement of organelles, cell migration and adhesion, endocytosis, differentiation, and ECM assembly [16,17]. Tubulin microtubules have a fundamental role in cell motility and division, in the transport of organelles and ciliary movement [16,17]. Vimentin intermediate filaments provide viscoelastic properties to the chondrocytes as well as signal transduction [16,17]. Lastly, the vinculin, (116-kDa) an actin-binding protein, plays an important function in cell adhesion and migration and it has a pleiotropic role in chondrocytic differentiation [18,19].

Our previous *in vitro* study investigated the effect of cyclical HP (1–5 MPa, 0.25 Hz) on actin and tubulin aspects of human normal and OA chondrocytes [20]. This study indicated that in OA chondrocytes cytoskeletal proteins were not well organized as well as in normal chondrocytes and, interestingly, showed that cyclical HP did not affect their distribution in OA cells.

The aim of this study was to examine the morphological aspects using TEM and SEM, and the organization of actin, tubulin, vimentin, and vinculin, by immunofluorescence (IF) technique, in cultured human normal and OA articular chondrocytes, exposed to IL-1β and cyclic HP.

## 2. Results

### 2.1. TEM and SEM Analysis

**Figure 1:**
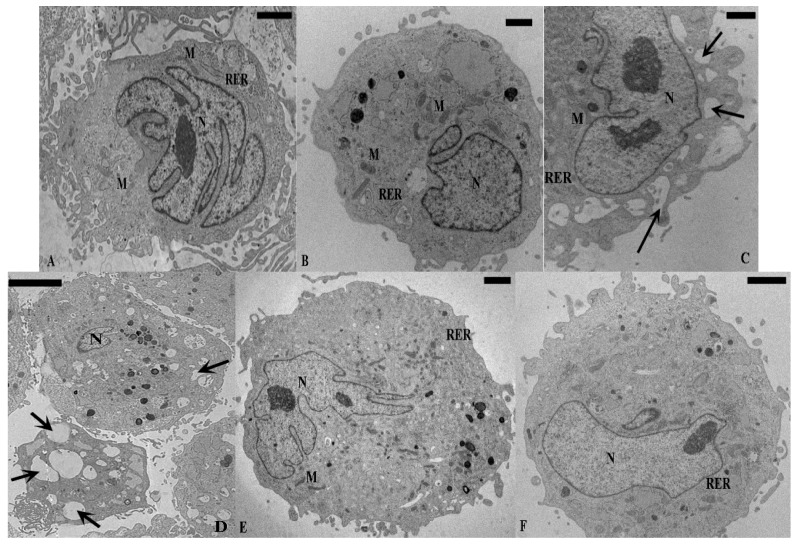

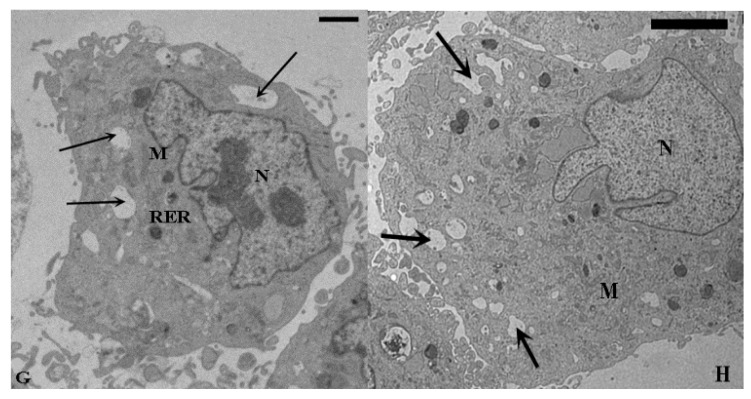
TEM micrographs of human cultured chondrocytes. Basal conditions: (**A**) Normal chondrocyte shows abundant rough endoplasmic reticulum (RER); the nucleus (N) contains normally-condensed chromatin; (**B**) OA chondrocyte displays euchromatic nucleus (N) and dilatation of the cisternae of rough endoplasmic reticulum (RER) in the cytoplasm. Incubation with IL-1β: normal (**C**) and OA chondrocytes (**D**); the cells present a cytoplasm with diffuse vacuolization (arrows) and contain a reduced amount of typical organelles such as rough endoplasmic reticulum (RER) and mitochondria (M). Cyclical hydrostatic pressure (HP): (**E**) normal chondrocyte maintains its shape and ultrastructure similar to basal conditions, nucleus (N) and rough endoplasmic reticulum (RER); (**F**) OA chondrocyte recovers many of the characteristic cytoplasmic structure, nucleus (N) and rough endoplasmic reticulum (RER). Exposure to HP+IL-1β: normal (**G**) and OA chondrocytes (**H**); the cells partially restore their morphology. The nucleus (N) appears euchromatic, the cytoplasm shows a restored organization with a reduced numbers of vacuoles (arrows), and mitochondria (M) are well shaped. (**A**,**F**,**G**) Bar: 5 µm, (**B**,**C**,**E**) Bar: 2 µm, (**D**,**H**) Bar: 10 µm.

**Figure 2:**
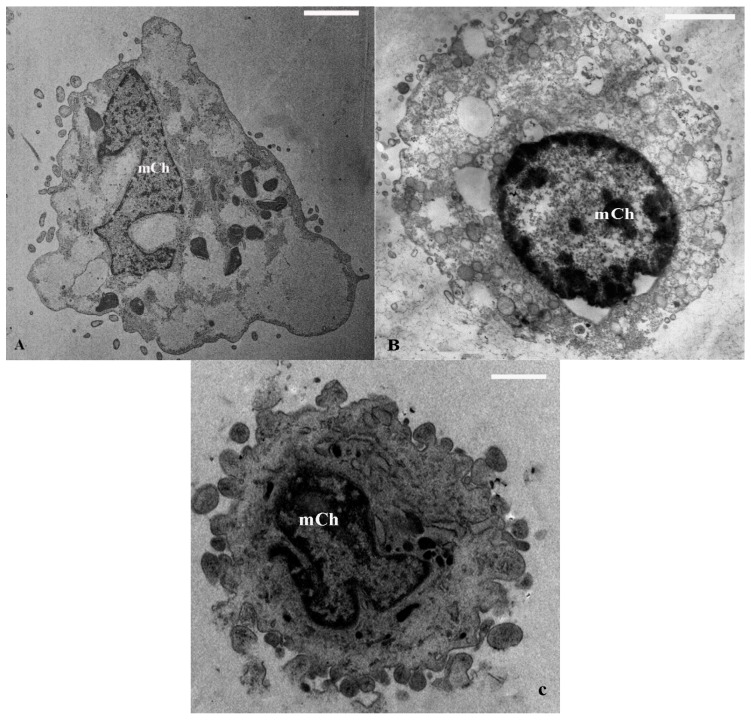
TEM images of marginated chromatin (mCh) in OA chondrocyte. Chromatin appears partially disrupted (**A**); condensed near the periphery of the cell (**B**); close to the nuclear envelope (**C**). Bar: 1 µm.

TEM analysis revealed some differences between normal and OA chondrocytes. Normal chondrocytes (Figure 1A) showed nuclei with euchromatic chromatin; in the cytoplasm the organelles were present and had a regular position: smooth endoplasmic reticulum and Golgi bodies were abundant, rough endoplasmic reticulum appeared rich in secretory material, and mitochondria were regularly shaped (Table 1). OA chondrocytes (Figure 1B) displayed a significant reduction in the number of mitochondria, and a significant increase in percentage of cells with vacuolization (≥5 vacuoles) and marginated chromatin, in comparison to normal cells (*p* < 0.01) (Table 1). Nuclei showed an enlarged and undulated shape and the chromatin appears partially disrupted, condensed near the periphery, close to the nuclear envelope (Figure 2).

Normal and OA chondrocytes exposed to with IL-1β contained several vacuoles in the cytoplasm (Figure 1C,D) and a reduced number of mitochondria and Golgi bodies in comparison to basal conditions (Table 1). Moreover, IL-1β significantly increased (*p* < 0.01) the percentage of normal and OA chondrocytes with marginated chromatin and vacuolization (Table 1). Cyclic HP did not change the morphology of normal chondrocytes (Figure 1E), but in OA cells partially restored many of the characteristic cytoplasmic structures (Figure 1F) and significantly increased the number of mitochondria in comparison to cells at basal conditions (*p* < 0.01) (Table 1). Normal and OA chondrocytes subjected to IL-1β and HP recovered in part the cytoplasmic ultrastructure (Figure 1G,H). In particular, OA chondrocytes showed a significant (*p* < 0.05) increase in the number of mitochondria and Golgi bodies and a significant decrease (*p* < 0.05) in the percentage of chondrocytes with vacuolization and marginated chromatin (*p* < 0.05), in comparison to cells treated with IL-1β alone (Table 1).

**Table 1:** Quantitative analysis of ultrastructural characteristics (TEM), in normal and OA chondrocytes, at different experimental conditions. Data are expressed as mean values ± SD.

Characteristics	Basal		IL-1β		Cyclical HP		Cyclical HP + IL-1β	
	N	OA	N	OA	N	OA	N	OA
Mitochondria (number per cell)	7.1 ± 2.5	3.3 ± 1.8 **	5.3 ± 1.1	2.6 ± 0.9	7.7 ± 2.7	6.6 ± 2.2 **	6 ± 1.4	4.1 ± 2.5 *
Golgi bodies (number per cell)	2.7 ± 1.2	1.7 ± 1.1	1.8 ± 1.2	0.8 ± 2.4	2.8 ± 1.1	2.7 ± 1.5	2.1 ± 1.3	1.9 ± 2.1 *
Vacuolization (>5) (cells %)	4.9 ± 1.2	13 ± 1.2 **	11 ± 1.1	32.6 ± 1.4 **	4.2 ± 0.10	9 ± 1.3	9 ± 1.2	20 ± 1.3 *
Marginated Chromatin (cells %)	5 ± 2.1	12 ± 1.7 **	10 ± 2.4	30 ± 2.7 **	5 ± 2.5	10 ± 1.9	7 ± 2.2	18 ± 1.8 *

TEM = transmission electron microscopy; N = normal chondrocyte; OA = osteoarthritic chondrocyte; IL-1β = interleukin-1β; HP = cyclical hydrostatic pressure; * *p* < 0.05: OA cyclical HP+IL-1β *vs.* OA IL-1β; ** *p* < 0.01: OA basal *vs.* normal basal; OA IL-1β *vs.* OA basal; OA cyclical HP *vs.* OA basal.

**Figure 3:**
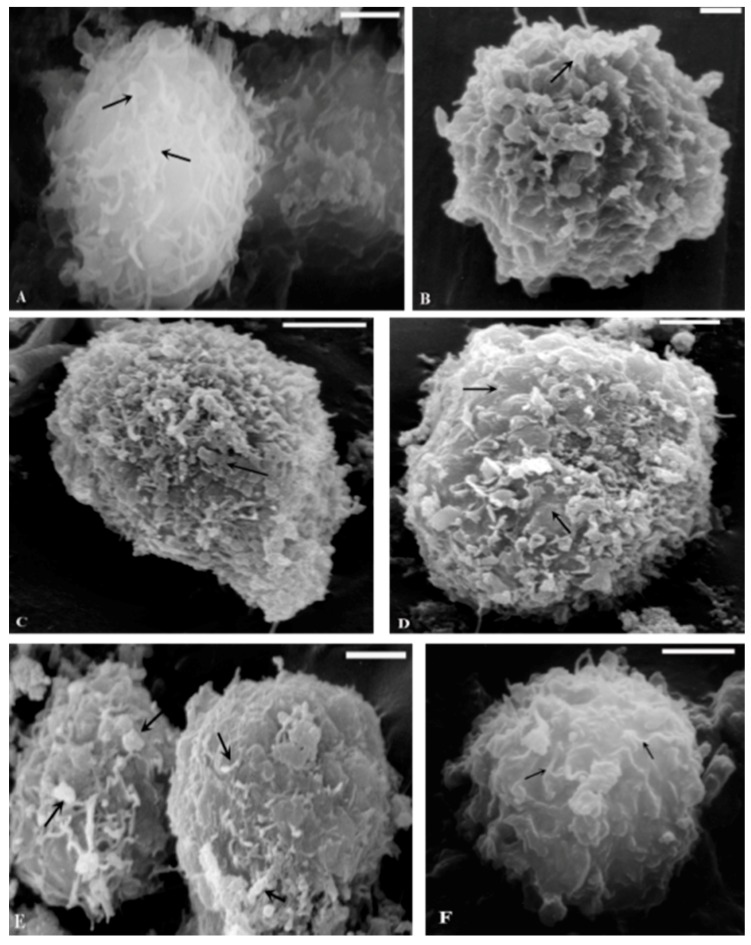
SEM micrographs of human cultured chondrocytes. Basal conditions: (**A**) normal chondrocyte shows many superficial process (arrows) and secretory granules; (**B**) OA chondrocyte presents spherical shape, containing some secretory granules (arrow), but lacking superficial process; Incubation with IL-1β: normal (**C**) and OA chondrocytes (**D**); the cells are devoid of granules and fibrils (arrows); Exposure to HP+IL-1β: normal (**E**) and OA chondrocytes (**F**); the cells show a spherical shape; granules and fibrils are detected (arrows). (**A**,**C**) Bar: 10 µm, (**B**) Bar: 2.5 µm, (**D**,**E**,**F**) Bar: 5 µm.

SEM examinations confirmed the results obtained by TEM. Under basal conditions, SEM images showed abundant matrix fibers and secretion granules in normal chondrocytes (Figure 3A), which were partially lost in OA chondrocytes (Figure 3B). On the contrary, both type of cells in presence of IL-1β exhibited a reduction of superficial processes and vesiculation (Figure 3C,D). SEM observations of normal chondrocytes subjected to HP did not display significant modifications compared to basal conditions, while OA cells partially acquired superficial characteristics similar to normal (Data not shown). Finally, normal and OA cells exposed to IL-1β and HP partially recovered surface morphology in comparison to cells treated with only IL-1β (Figure 3E,F).

### 2.2. Immunocytochemical Examination

Immunocytochemical examination of cytoskeleton components in human normal and OA chondrocytes under different experimental conditions was also reported.

**Figure 4:**
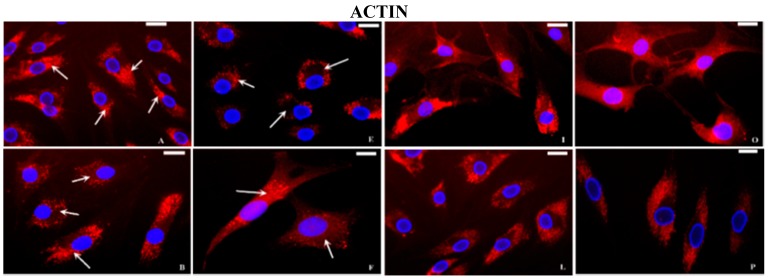

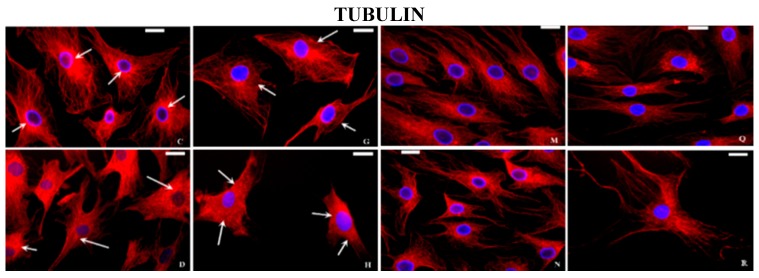
Indirect immunofluorescence microscopy. Basal conditions: (**A**) normal chondrocytes incubated with monoclonal anti-β-actin antibody show intense fluorescence at the polarity of the cytoplasm (arrows); OA chondrocytes incubated with monoclonal anti-β-actin antibody show a not-well-defined and polarized signal (arrows) (**B**); normal chondrocytes incubated with monoclonal anti β-tubulin antibody show a signal diffused from the nucleus to the periphery of the cytoplasm (arrows) (**C**); in OA chondrocytes (**D**) the signal is present but fragmented (arrows). Incubation with IL-1β: Normal and OA chondrocytes show a reduction of the fluorescence and an altered distribution of the actin filaments (**E**, **F** respectively, arrows) and disassembled tubulin filaments (**G**, **H** respectively, arrows). Cyclical hydrostatic pressure (HP): the localization of actin (**I**, **L** respectively) and tubulin (**M**, **N** respectively) proteins in normal and OA chondrocytes appear similar to basal conditions. Exposure to HP+IL-1β: normal and OA chondrocytes incubated with anti-β-actin (**O**, **P** respectively) and anti-β-tubulin (**Q**, **R** respectively) antibodies show a recovery in the organization of both filaments in comparison to the cells stimulated with IL-1β alone. Nuclei (blue) were stained with DAPI and anti-β-actin and anti-β-tubulin (red). Bar: 50 μm.

**Figure 5:**
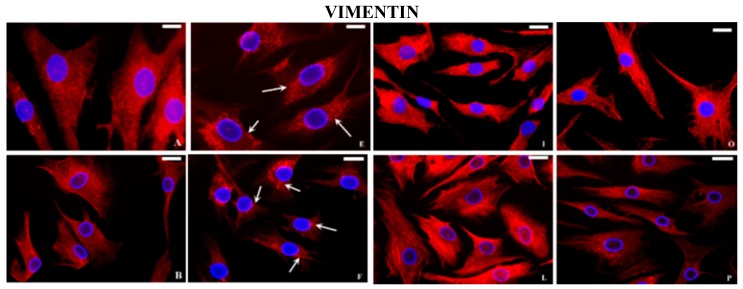

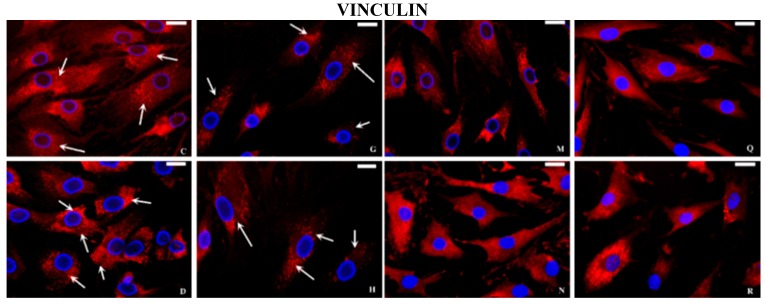
Indirect immunofluorescence microscopy. Basal conditions: the vimentin filaments in normal chondrocytes (**A**) were organized in all the cytoplasm as a network, crossing from the cell periphery to the nuclear membrane; in OA cells (**B**) the distribution is partially altered. Normal (**C**) and OA chondrocytes (**D**) incubated with anti-vinculin antibody show a punctate pattern under the plasma membrane (arrows). Incubation with IL-1β: normal and OA chondrocytes show a reduced fluorescence and a destruction of both vimentin (**E**, **F** respectively, arrows) and vinculin (**G**, **H** respectively, arrows) filaments. Cyclical hydrostatic pressure (HP): fluorescent signal of vimentin and vinculin proteins in normal (**I**, **M** respectively) and OA chondrocytes (**L**, **N** respectively). Exposure to HP + IL-1β: fluorescent signal of vimentin and vinculin proteins in normal (**O**, **Q** respectively) and OA chondrocytes (**P**, **R** respectively). Nuclei (blue) were stained with DAPI and anti-vimentin and anti-vinculin (red). Bar: 50 μm.

At basal conditions, fluorescent microscopy analysis showed a clear polarization of the actin signal on the apical sides of the cytoplasm in normal chondrocytes (Figure 4A). On the contrary, in OA cells the actin localization was not well-defined but diffused in the cytoplasm or limited to the periphery of the cells (Figure 4B). The tubulin protein was organized in filaments from the nucleus to the periphery of the cytoplasm in normal chondrocytes (Figure 4C) whereas in OA cells, filamentous structure of tubulin appeared partially lost (Figure 4D). In normal chondrocytes, the vimentin filaments were organized in all the cytoplasm as a network, crossing from the cell periphery to the nuclear membrane (Figure 5A), in OA they appeared disorganized (Figure 5B). The spots of vinculin were clearly defined and revealed a punctated pattern under the plasma membrane in normal (Figure 5C) and OA chondrocytes, even if in OA cells the interaction with actin filaments was less evident (Figure 5D). After stimulation with IL-1β, in normal and OA chondrocytes, actin (Figure 4E,F), tubulin (Figure 4G,H), vimentin (Figure 5E,F), and vinculin (Figure 5G,H), appeared differently distributed and disassembled in comparison to basal conditions. The distribution of actin and tubulin proteins in human normal and OA chondrocytes subjected to HP alone was similar to that observed at basal conditions (Figure 4I,L,M,N). In a similar manner, vimentin (Figure 5I) and vinculin (Figure 5M) organization did not appear changed in normal chondrocytes, subjected to HP, whereas the filamentous structure of vimentin (Figure 5L) and the relationship between vinculin and actin (Figure 5N) were ameliorated in OA chondrocytes compared to basal conditions.

Finally, HP used in our research counteracted the negative actions of IL-1β on cytoskeletal proteins distribution in normal (Figure 4O actin; Figure 4Q tubulin; Figure 5O vimentin; and Figure 5Q vinculin) and OA chondrocytes (Figure 4P actin; Figure 4R tubulin; Figure 5P vimentin; and Figure 5R vinculin).

## 3. Discussion

In this study we investigated by TEM, SEM, and IF techniques the cellular morphology and the organization of actin, tubulin, vimentin, and vinculin in normal and OA human chondrocytes exposed to IL-1β and/or cyclical low HP. For this purpose the employed pressure was close to the physiological range of the human joint; in fact, pressure levels of 5 MPa are measured in the knee joint during normal gait [21]. Furthermore, in our study we applied HP for a time as short as possible (3 h) to approximate physiological conditions of the human joint.

Previous studies reported significant structural differences between human normal and OA chondrocytes at the nuclear, cytoplasmic levels and in the organization of actin and tubulin filaments [11,20,22]. Our current results confirmed that in OA chondrocytes the number of mitochondria and Golgi bodies was reduced, compared to normal chondrocytes. The morphological aspects of OA chondrocytes showed signs of cellular suffering with an increase of the percentage of cells rich in vacuolization and in marginated chromatin. The addition of IL-1β induced serious morphological modifications in normal and OA chondrocytes which were counteracted by HP. The recovery of the number of cytoplasmic organelles after HP exposition is in agreement with previous observations, demonstrating an increase of metabolic activity in chondrocytes subjected to cyclical, low pressurization [12,20,23].

Marginated chromatin is reported to be a peculiar ultrastructural feature of apoptosis; apoptosis, necrosis, chondroptosis, or combinations of these processes have been implicated in the pathogenetic pathway of OA [24,25,26]. Different studies demonstrated a reduced number of chondrocytes due to an increase of the percentage of apoptosis in OA cartilage, with a potential link to IL-1β as the possible trigger in these processes [27,28]. Heraud *et al.* [29] described that in human OA cartilage, 18%–21% of chondrocytes presented apoptotic features and that IL-1β increased, in a dose-dependent manner, the percentage of apoptotic cells in both normal and OA cartilage. In agreement with the previous observations, we found that IL-1β induced a significant increase of the percentage of OA cells with marginated chromatin, typical evidence of apoptosis [24,25]. How IL-1β induces the death of chondrocytes is not entirely understood, but NO has been strongly suggested as a possible mediator [4].

Cyclical low HP used in our study reduced, in a significant (*p* < 0.05) manner, the percentage of cells with marginated chromatin induced by IL-1β in OA chondrocytes. Loading effects on chondrocyte apoptosis have been widely studied *in vitro* [30,31,32]; however, these studies have generated contrasting results due to experimental variation, namely in the tissue evaluated (species, age, anatomical location) and test conditions used (magnitude, frequency and time of pressure, and the mechanism used to apply pressure).

Modifications of the cytoskeletal organization in chondrocytes were described in the course of OA. These alterations affect a series of phenomena, including cellular differentiation and proliferation, formation and flux of vesicles, synthesis of the ECM molecules, adhesion, and cellular migration [33,34,35,36,37].

The present results of IF, with anti-actin and anti-tubulin antibodies, confirmed the data obtained in our previous investigations [20,22] regarding the different localization of the two proteins and the different organization between normal and OA chondrocytes. Actin and tubulin cytoskeletal elements have been extensively studied in chondrocytes, but less is known about the organization of intermediate filaments, vimentin and vinculin [13,38,39,40]. In this study, the filament network organization of vimentin was altered in OA chondrocytes, in comparison to normal chondrocytes, according to other authors [39,40]. The vimentin intermediate filament network is involved in the maintenance of the chondrocyte phenotype, and may play a role in the mechanotransduction pathways; changes in vimentin organization contributed to development and progression of OA [40]. Vinculin forms a part of a macromolecular complex on the cytoplasmic face of integrin-mediated cellular junctions with the ECM; therefore, vinculin has pleiotropic roles in chondrogenesis, and regulates the expression of chondrocyte-specific genes via the integration of various signaling pathways [16]. In our study the spots of vinculin were clearly defined in normal and OA chondrocytes, but in OA cells the interaction with actin filaments was less evident.

It would be of great interest to establish whether the cytoskeletal modifications described lead to OA disease or if the changes of cytoskeleton are a result of OA. In this study, we used IL-1β to create an *in vitro* model reproducing the circumstances leading to *in vivo* cartilage degradation in OA [6]; this negative stimulus, affected the cytoskeletal proteins in both normal and OA chondrocytes. Interestingly, IL-1β has also been shown to increase F-actin amounts in chondrocytes and seems to be involved in the regulation of many cytoskeleton-related genes suggesting that a finely-balanced interplay of cytokines may exist to regulate cytoskeletal element dynamics and hence organization [41,42].

Little is known about how the chondrocyte cytoskeleton responds to mechanical loading because *in vitro* models presented different experimental culture conditions [20,43,44,45,46,47]. As reported in the present study, exposure to low magnitude cyclic HP did not change the organization of actin and tubulin in normal and OA chondrocytes, confirming our previous results [20]. In this paper we also investigated intermediate filaments, demonstrating that HP alone did not influence the vimentin and vinculin structure in normal chondrocytes, but appears to improve their organization in OA cells. Furthermore, this pressure seems to be able to counterbalance the negative effect of IL-1β on cytoskeletal proteins in normal and OA chondrocytes. It should be interesting to evaluate if HP could recover the cytoskeleton organization after incubation with agents that promote the dissolution of microfilaments (cytochalasin B) or microtubules (nocodazole).

The exact mechanism of action of HP on chondrocytes has not yet been fully elucidated. Mechanical stimulation of chondrocytes leads to ion flux across the cell membrane, which converts the physical stimulus to a chemical signal [48]. Activation of ion channels allows an influx of ions, such as calcium, into the cells that leads to the activation of intracellular signaling pathways. In particular, several studies have indicated that HP application on chondrocytes determined an increase of intracellular calcium dependent on the direct effects of HP on stretch-activated calcium channels, as well as the release from intracellular stores [49,50]. Furthermore, intermittent HP in chondrocyte cell culture systems results in concurrent increase of cyclical adenosine monophosphate (cAMP) [51]. However, the contribution of these signaling events in the ultrastructure and cytoskeletal organization observed in our study must still be clarified. Furthermore, we have to underline the high sensitivity of OA chondrocytes in comparison to normal cells to HP as reported by other investigators [12,52].

In conclusion, our results confirmed structural differences at nuclear, cytoplasmic and cytoskeletal level between normal and OA chondrocytes. IL-1β induced ultrastructural and cytoskeletal modifications, counteracted by a cyclical low HP corresponding to the physiological pressure in human joints. Nevertheless, the present study has several limitations which warrant mention. Firstly, the use of monolayer cultures, even if HP showed to act on cytoskeletal organization, in similar models of chondrocyte cultures [33,45]. Secondly, other limitations concern the lack of additional techniques, such as Western blot analysis, to confirm changes in amounts of cytoskeletal proteins and the lack of immunogold labeling to identify cytoskeletal proteins by TEM. Finally, considering that the cytoskeleton is mechanically coupled to other cells and ECM via transmembrane integrins, it remains to be determined the possible effect of HP on integrin behavior and the relationship with the observed modifications on cytoskeleton proteins [53].

More sophisticated experiments will be necessary to analyze the effects of HP on chondrocytes morphology and metabolism in order to clarify the effective role of mechanical factors in the etiopathogenesis of OA and the importance of physical activity in prevention and treatment of OA patients.

## 4. Experimental Section

### 4.1. Materials

Dulbecco’s Modified Eagle Medium (DMEM), penicillin, streptomycin, amphotericin B, fetal calf serum, glutamine, phosphate-buffered saline (PBS), bovine serum albumin (BSA) and normal goat serum (NGS) were purchased from GIBCO/Invitrogen (Grand Island, NE, USA). Hyaluronidase, pronase, collagenase, interleukin (IL)-1β, Trypan blue solution, mouse monoclonal anti-β-tubulin and mouse monoclonal anti-vinculin antibodies were purchased from Sigma-Aldrich (Milano, Italy). Methanol and acetone were obtained from Società Italiana Chimici (Roma, Italy). Surlyn 1801 Bynel CXA 3048 bilayer membrane (thickness 90 μm) were purchased from Du Pont (Milan, Italy) and Jet Melt 3764 adhesive from 3M (Milan, Italy). Mouse monoclonal anti-β-actin antibody and mouse monoclonal anti-vimentin antibody were purchased from Santa Cruz Biotechnology (Dallas, TX, USA), while goat anti-mouse IgG-Texas Red conjugated antibody from Southern Biotechnology (Birmingham, AL, USA). Vectashield were purchased from Vector Labs (Burlingame, CA, USA) while staining: 4′,6-diamidino-2-phenylindole (DAPI) was purchased from Invitrogen, Molecular Probes (Monza, Italy).

### 4.2. Cell Culture

Normal human articular cartilage was obtained from femoral heads of five subjects (three males and two females) with displaced femoral neck fractures; OA human articular cartilage was obtained from the femoral heads of five patients (three males and two females) with hip OA defined by clinical and radiological ACR criteria [54] undergoing total hip replacement surgery. The mean age of the group was 63 years (range: 49–70) for normal subjects and 68 years (range 63–74) for OA patients. The study protocol was approved by the Ethics Committee of the Azienda Ospedaliera Universitaria Senese, Siena, Italy (decision No. 726/07). Each participant in this study signed a written consent. Only cartilage samples from unique donors were employed for each culture. Thus, for each experiment, cells come from the same subject.

Normal chondrocytes were acquired from the middle layer of femoral heads cartilage, whereas OA chondrocytes originated from the area adjacent to the OA lesion. OA cartilage was not macroscopically altered; after a histological analysis of representative samples showed typical osteoarthritic changes such as the presence of chondrocyte clusters, loss of metachromasia, and fibrillation (Mankin degree 3–7) [55]. Normal cartilage was characterized by a glossy, white, completely smooth surface and a healthy appearance without irregularities.

After surgery, the cartilage was aseptically dissected and was cut into small pieces. The fragments were washed in DMEM with phenol red, containing 2% penicillin/streptomycin solution and 0.2% amphotericin B. The chondrocytes were obtained from the articular cartilage using sequential enzymatic digestion: 30 min with 0.1% hyaluronidase, 1 h with 0.5% pronase, and 1 h with 0.2% collagenase at 37 °C in the wash solution (DMEM supplemented with penicillin/streptomycin solution and amphotericin B). The resulting cell suspension was filtered twice using 70-μm nylon meshes, then washed and centrifuged for 10 min at 700 g. Trypan blue viability test showed that 90%–95% of the recovered cells were alive.

Cells were incubated at 37 °C and 5% CO_2_ in culture medium (DMEM + 10% fetal calf serum + 200 U/mL penicillin + 200 U/mL streptomycin) for two weeks. The medium was changed three times per week. The cell morphology was examined daily under an inverted microscope (Olympus IMT-2, Kumamoto, Japan) to avoid the dedifferentiation of expanded chondrocytes and to preserve their phenotypic stability. In the first passage normal and OA human chondrocytes were seeded in Petri dish (35 mm × 10 mm) at a starting density of 6 × 10^4^ cells and overlay with 2 mL of medium with phenol red composed by 10% fetal calf serum, 200 U/mL penicillin, 200 U/mL streptomycin, and 2 mM glutamine until they became confluent.

The primary cultures of chondrocytes were evaluated at basal conditions, after stimulation with IL-1β (5 ng/mL) for 48 h with or without cyclic HP. After all treatments the cells were processed for TEM, SEM, and IF.

### 4.3. Pressurization System

Our pressurization system presents some special characteristics already described in detail [56]. In the present study the chondrocytes cultivated on Petri dishes were exposed to cyclic pressurization by sinusoidal waves (minimum pressure 1MPa and maximum pressure 5 MPa) at 0.25 Hz frequency for a 3 h period. Some dishes were cultivated in the loading chamber without receiving any pressurization, and used as controls.

### 4.4. Morphological Analysis

For TEM examination, cell samples were fixed in cold Karnovsky fixative [57] and maintained at 4 °C for 2 h. Fixed chondrocytes was washed in 0.1 mol/L cacodylate buffer (pH 7.2) for 12 h, postfixed in 1% buffered osmium tetroxide for 1 h at 4 °C, then dehydrated in a graded ethanol series, and embedded in Epon–Araldite. Ultra-thin sections were cut with a Supernova ultramicrotome (Reickert Jung, Vienna, Austria), mounted on copper grids, stained with uranyl acetate and lead citrate, and then observed and photographed with a Philips EM 208 transmission electron microscope (Philips Scientifics, Eindhoven, The Netherlands). At least 100 chondrocytes from each group we evaluated.

### 4.5. Scanning Electron Microscope Analysis

An aliquot from the same cell samples was also processed for SEM, fixing the chondrocytes as described above, and smearing them on polylysine (1%)-coated cover slides. After dehydration, specimens were dried by the critical point technique, coated in gold, and examined with a Philips 505 scanning electron microscope (Philips Scientifics, Eindhoven, The Netherlands). At least 200 chondrocytes from each group were evaluated.

### 4.6. Immunofluorescence Microscopy

Immunocytochemical investigations on the organization and distribution of cytoskeletal proteins were performed in chondrocytes grown on coverslips in medium with phenol red composed by 10% fetal calf serum, 200 U/mL penicillin, 200 U/mL streptomycin, and 2 mM glutamine, at a density of 1 × 10^5^ cell/mL. After treatments, the cells were washed in PBS and fixed in methanol and in acetone for 15 and five minutes at −20 °C, respectively. Subsequently the samples were saturated for 20 min at room temperature with PBS–BSA (1%) containing NGS (5%) and then incubated overnight at 4 °C with an anti-β-actin, anti-β-tubulin, anti-vimentin, and anti-vinculin mouse monoclonal antibodies, diluted 1:100, 1:100, 1:200, and 1:800, respectively, in PBS/0.1% BSA/1% NGS. The reaction was revealed by a goat anti-mouse IgG-Texas Red conjugated antibody diluted 1:100 in PBS/0.1% BSA/1% NGS, for 1 h at room temperature. Finally, the samples were washed three times in PBS and the coverslips mounted with Vectashield. Incubation with primary antibody was omitted in control samples. Nuclei were stained with 1 μg/mL DAPI for 1 min after removal of secondary antibodies. Fluorescence was observed with Leitz Aristoplan light microscope (Leica, Wetzlar, Germany) equipped with a fluorescence apparatus. Images were acquired and analyzed with Leica software. At least 100 chondrocytes from each group were evaluated [20].

### 4.7. Morphometric and Statistical Analysis

For the morphometric studies, we analyzed sections of three different blocks from each group. For standardization and comparison of the different groups, only medially-sectioned chondrocytes were investigated; 100 chondrocytes were selected using the nucleus/cytoplasm ratio as the selection criterion. Our analysis was based on an established method for ultrastructural quantitative evaluation of changes in chondrocytes [11,58]. Mitochondria and Golgi bodies were counted and expressed the mean ± standard deviation (SD) of triplicate values for each experiment. The presence of cytoplasmic vacuolization was reported as percentages of the cells with a number ≥ of five vacuoles, considered as point of reference [59]. χ^2^ test was used to analyze the percentage of vacuolization and the percentage of cells with marginated chromatin [24,25]. Immunocytochemistry staining intensity was scored by the same researcher and expressed as percentages of the total number of cells showing a regular distribution after incubation with the specific antibody for each studied protein [20,22].

Analysis of variance followed by the Bonferroni multiple comparison test was used to make comparisons. All analyses were performed using the SAS System (SAS Institute Inc., Cary, NC, USA) and GraphPad Prism. A *p* value <0.05 was accepted as statistically significant.
